# Effects of four disease-controlling agents (chlorothalonil, CuCl_2_, harpin, and melatonin) on postharvest jujube fruit quality

**DOI:** 10.1038/s41598-023-35392-1

**Published:** 2023-05-22

**Authors:** Shan Tian, Ying Chen, Qianjin Wang, Zhilan Liu, Yueyue Li, Xusheng Zhao

**Affiliations:** 1 Life Science College, Luoyang Normal University, Luoyang, 471934 Henan China; 2 Grain and Oil Crops Technology Extension Station, Yongchuan, 402160 Chongqing China

**Keywords:** Plant physiology, Plant sciences

## Abstract

Postharvest senescence and disease development can reduce the nutritional value of fresh jujube fruit. Herein, four different disease-controlling agents (chlorothalonil, CuCl_2_, harpin and melatonin) were separately applied to fresh jujube fruit, and all improved postharvest quality (evaluated by disease severity, antioxidant accumulation and senescence) relative to controls. Disease severity was drastically inhibited by these agents, in the order chlorothalonil > CuCl_2_ > harpin > melatonin. However, chlorothalonil residues were detected even after storage for 4 weeks. These agents increased the activities of defense enzymes including phenylalanine ammonia-lyase, polyphenol oxidase, glutathione reductase and glutathione S-transferase, as well as accumulation of antioxidant compounds such as ascorbic acid, glutathione, flavonoids and phenolics, in postharvest jujube fruit. The enhanced antioxidant content and antioxidant capacity (evaluated by Fe^3+^ reducing power) was ordered melatonin > harpin > CuCl_2_ > chlorothalonil. All four agents significantly delayed senescence (evaluated by weight loss, respiration rate and firmness), with the effect ordered CuCl_2_ > melatonin > harpin > chlorothalonil. Moreover, treatment with CuCl_2_ also increased copper accumulation ~ threefold in postharvest jujube fruit. Among the four agents, postharvest treatment with CuCl_2_ could be considered most appropriate for improving postharvest jujube fruit quality under low temperature conditions without sterilization.

## Introduction

Jujube (*Zizyphus jujuba* cv.), indigenous to China, has been consumed for > 4000 years, and > 700 cultivars have been identified^1^. Jujube fruit is commonly utilized in food products (paste, puree, and confectionary) and additives due to its high nutritional value^2,3^. Greater accumulation of bioactive components (e.g., vitamin C and phenolics) has been detected in unripe jujube fruit compared with ripe fruit^4^. However, jujube is a non-climacteric fruit, and its quality markedly decreases upon storage at room temperature for ~ 1 week^5^. Within the supply chain, postharvest losses in quality of fresh jujube can be attributed to water loss, browning, softening, alcoholic fermentation, and even decomposition^6^. This is a serious problem in terms of extending storage life and improving quality values (e.g., nutrition, aroma, and taste). Many methods have been developed to delay postharvest fruit and vegetable senescence^7–9^. For example, treatment with an essential mineral mixture has been shown to induce antioxidant accumulation and delay postharvest senescence in jujube fruit^8^. Moreover, recent research showed that methods combining chemical treatments with biological control strategies or physical techniques consistently achieved better preservation quality compared with individual treatment^6^.

Fruit ripening and senescence are associated with increased susceptibility to postharvest pathogens, and the traditional approach for decay control in fruit is to use fungicides^10^. In general, pesticides kill or damage pathogens and pests, and protect crops in the field as well as during postharvest storage. For example, organic pesticides such as chlorothalonil (CHT), which binds to the thiol-rich enzyme glyceraldehyde-3-phosphate dehydrogenase of pathogens^11^, are commonly used during grapevine growth to improve the quantity and quality of grapes^12^. To alleviate pesticide toxicity, glutathione reductase (GR) and glutathione S-transferase (GST), coupled with glutathione (GSH), are required for chlorothalonil degradation in plant leaves^13^. However, the use of high concentrations of organic pesticide poses a great threat to both human health and the environment^14^. By contrast, copper, the major component of Bordeaux mixture (an inorganic pesticide), can induce hydroxyl radical formation via Fenton reaction^15^, delay cut flower senescence, and it has been widely used to control various pathogens in agriculture^16,17^.

Many elicitors are applied to plants to control disease development^18^. Interestingly, reports have shown melatonin can enhance germination, delay postharvest senescence, improve abiotic stress tolerance, and protect against a broad array pathogens of plants^19–21^. Regarding the underlying mechanism, melatonin may modify plant growth and development by acting as an antioxidant and/or affecting expression of related genes^19^. Harpin, a well-characterized elicitor isolated from *Erwinia amylovora*, also delays postharvest senescence in plants^22,23^, induces hypersensitive responses, and enhances resistance against various pathogens^24^ Moreover, induction of phenylalanine ammonia-lyase (PAL) and polyphenoloxidase (PPO) activities is required for harpin-induced rust disease resistance in winter jujube^25^. Interestingly, harpin is believed to induce defense-related gene expression and activate antioxidant metabolite biosynthesis in plants^22^.

These disease control agents can inhibit pathogen development and influence antioxidant metabolism in plants to varying degrees^11,16,19,22^. Interestingly, these agents can be divided into four groups: organic pesticides (CHT), inorganic pesticides (CuCl_2_), biological pesticides (harpin) and phytohormones (melatonin). Here, we investigated which agent may be the best choice for maintaining the quality of postharvest jujube fruit. The four agents (CHT, CuCl_2_, harpin and melatonin) were separately applied to postharvest jujube fruit without sterilization, their effects on disease resistance (natural infection), antioxidant accumulation, and senescence level were investigated under low temperature (4 °C), and the potential underlying mechanisms were explored. The findings provide insight into disease-controlling agents regulating postharvest jujube fruit quality from an antioxidant nutrient perspective.

## Results

### Disease indices and defense enzyme activities

The disease index in postharvest jujube fruit was ordered control > melatonin > harpin > CuCl_2_ > CHT. Application of CHT, CuCl_2_, harpin, and melatonin reduced the disease index by ~ 78%, ~ 68%, ~ 54%, and ~ 41%, respectively, after storage for 28 days, relative to controls (Table 1; *p* < 0.05). Compared with controls, treatment with CuCl_2_ reduced the disease index in jujube fruit by ~ 80%, 74%, 70%, and 68% after storage for 7, 14, 21, and 28 days, respectively.

**Table 1:** Disease index in jujube fruit.

	Day 7	Day 14	Day 21	Day 28
Controls	22.8 ± 1.7^a^	38.2 ± 2.3^a^	58.2 ± 4.7^a^	80.5 ± 2.5^a^
CHT	2.3 ± 0.5^e^	6.5 ± 1.1^e^	12.1 ± 1.7^e^	17.8 ± 1.3^e^
CuCl_2_	4.5 ± 0.4^d^	9.8 ± 1.3^d^	17.2 ± 1.4^d^	25.7 ± 3.7^d^
Harpin	8.3 ± 0.8^c^	15.7 ± 1.3^c^	24.2 ± 3.5^c^	37.4 ± 2.8^c^
Melatonin	13.2 ± 1.4^b^	22.5 ± 2.5^b^	34.8 ± 3.1^b^	47.5 ± 4.2^b^

Effects of chlorothalonil, CuCl_2_, harpin, and melatonin on disease index (%) monitored in jujube fruit after storage for 7, 14, 21, and 28 days at 4 °C. Means associated with the same letter are not significantly different for each day (*n* = 3; *p* < 0.05).

**Figure 1:**
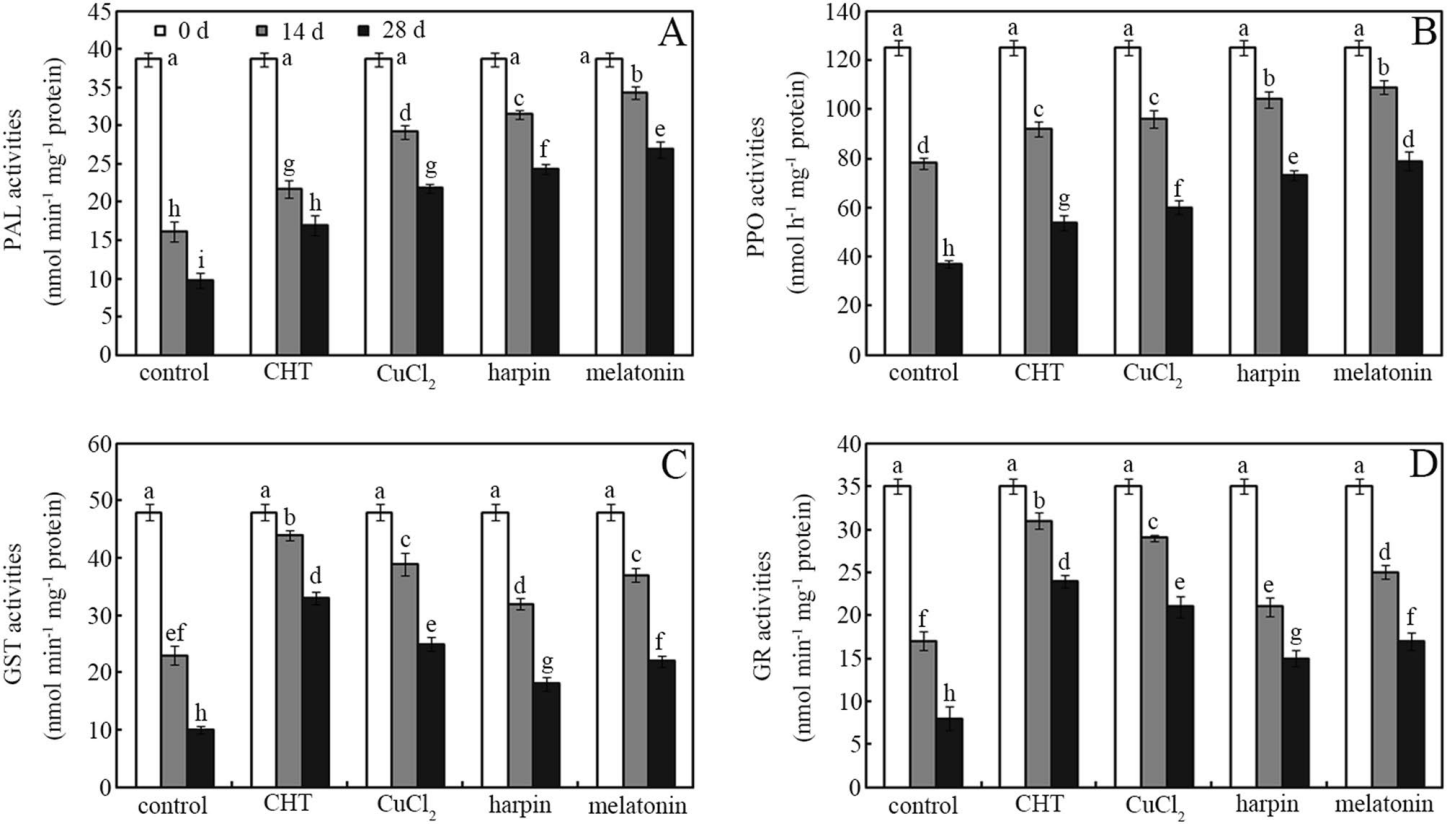
Enzyme activities of postharvest jujube fruit. Effects of chlorothalonil (CHT), CuCl_2_, harpin and melatonin on PAL (**A**), PPO (**B**), GST (**C**) and GR (**D**) activities were monitored in jujube fruit after storage for 0, 14 and 28 days at 4 °C. Bars represent standard deviation of the mean (*n* = 3); means associated with the same letter are not significantly different (*p* < 0.05).

PAL and PPO activities in postharvest jujube fruit were ordered melatonin > harpin > CuCl_2_ > CHT > controls. Application of CHT, CuCl_2_, harpin and melatonin enhanced PAL activities by ~ 74%, ~ 125%, ~ 151% and ~ 177%, respectively, in jujube fruit after storage for 28 days, relative to controls (Fig. 1a; *p* < 0.05). Similar pattern changes were also observed for PPO activities (Fig. 1b; *p* < 0.05). Application of CHT, CuCl_2_, harpin, and melatonin enhanced PPO activities in jujube fruit by ~ 18%, ~ 23%, ~ 33%, and ~ 40%, respectively, after storage for 14 days, relative to controls (Fig. 1b; *p* < 0.05).

By contrast, GST and GR activities in postharvest jujube fruit were ordered CHT > CuCl_2_ > melatonin > harpin > controls. Application of CHT, CuCl_2_, harpin, and melatonin enhanced GST activities in jujube fruit by ~ 91%, ~ 70%, ~ 39%, and ~ 61%, respectively, after storage for 14 days, relative to controls (Fig. 1c; *p* < 0.05). Similar pattern changes were also observed for GR activities (Fig. 1d; *p* < 0.05). Application of CHT, CuCl_2_, harpin, and melatonin enhanced GR activities in jujube fruit by ~ 200%, ~ 163%, ~ 88% and ~ 113%, respectively, after storage for 28 days, relative to controls (Fig. 1d; *p* < 0.05).

### Antioxidant accumulation and antioxidant capacity in jujube fruit

**Figure 2:**
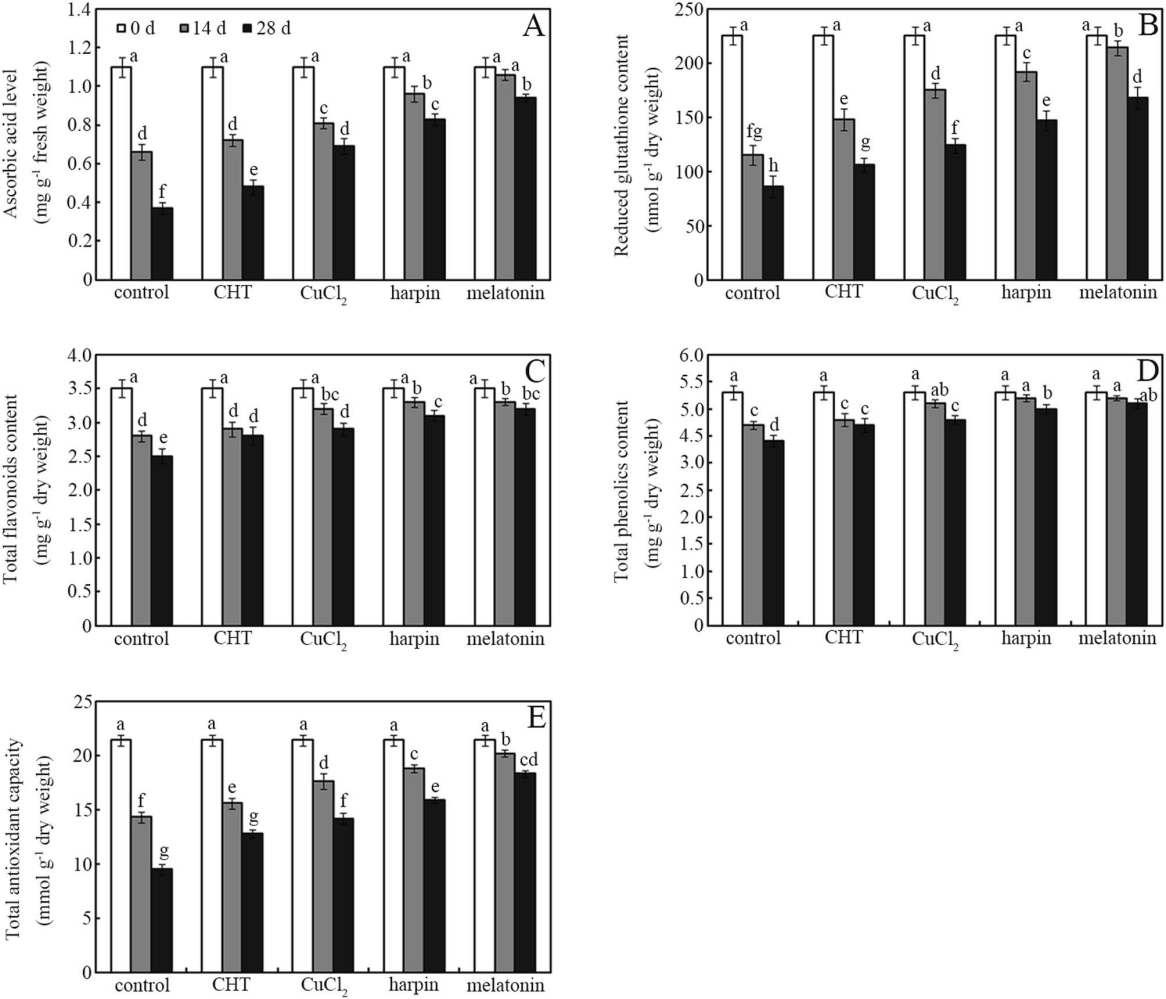
Antioxidant nutrient and total antioxidant capacities. Effects of chlorothalonil (CHT), CuCl_2_, harpin and melatonin on ascorbic acid (**A**), reduced glutathione (**B**), total flavonoids (**C**) and total phenolics levels (**D**), and total antioxidant capacity (E), were monitored in jujube fruit after storage for 0, 14 and 28 days at 4 °C. Bars represent standard deviation of the mean (*n* = 3); means associated with the same letter are not significantly different (*p* < 0.05).

Application of CHT, CuCl_2_, harpin and melatonin significantly enhanced levels of ascorbic acid, glutathione and total phenolics, coupled with enhanced total antioxidant capacity, in jujube fruit after storage for 14 and 28 days (Fig. 2; *p* < 0.05). Treatment with harpin increased ascorbic acid, glutathione, total flavonoids and total phenolics levels in jujube fruit by ~ 124%, ~ 71%, 24%, and ~ 18%, respectively, after storage for 28 days (Fig. 2a–d; *p* < 0.05). Moreover, antioxidant accumulation and antioxidant capacity in postharvest jujube fruit were ordered melatonin > harpin > CuCl_2_ > CHT > controls. Application of CHT, CuCl_2_, harpin, and melatonin enhanced ascorbic acid in jujube fruit by ~ 9%, ~ 23%, ~ 45%, and ~ 61%, respectively, after storage for 14 days, relative to controls (Fig. 2a; *p* < 0.05). Similarly, treatment with CHT, CuCl_2_, harpin, and melatonin elevated total antioxidant capacity in jujube fruit by ~ 35%, ~ 49%, ~ 67% and ~ 93%, respectively, after storage for 28 days, relative to controls (Fig. 2e; *p* < 0.05).

### H_2_O_2_ content

**Figure 3:**
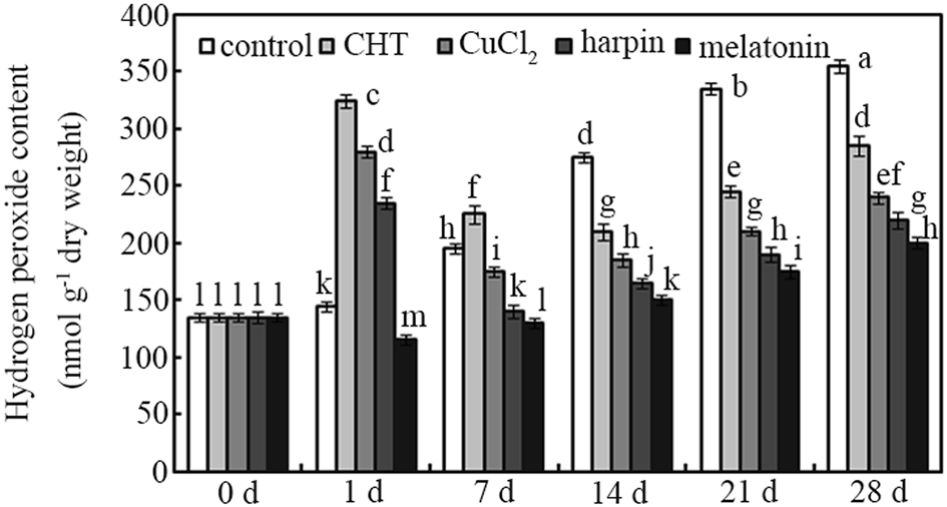
H_2_O_2_ content of postharvest jujube fruit. Effects of chlorothalonil (CHT), CuCl_2_, harpin and melatonin on H_2_O_2_ content were monitored in jujube fruit after storage for 0, 7, 14, 21 and 28 days at 4 °C. Bars represent standard deviation of the mean (*n* = 3); means associated with the same letter are not significantly different (*p* < 0.05).

Compared with day 0, H_2_O_2_ content was changed significantly in postharvest jujube fruit during the first 28 days (Fig. 3; *p* < 0.05). H_2_O_2_ content was increased by 7.4%, 44.4%, 103.7%, 148.1% and 163% in the water control group after storage for 1, 7, 14, 21 and 28 days, respectively (Fig. 3; *p* < 0.05). After treatment for 1 day, H_2_O_2_ levels in postharvest jujube fruit were ordered CHT > CuCl_2_ > harpin > control > melatonin. Compared with controls, during day 1, H_2_O_2_ accumulation in postharvest jujube fruit increased after treatment with CHT (~ 124%), CuCl_2_ (~ 93%), and harpin (~ 62%), but decreased for melatonin (~ 21%) (*p* < 0.05). Thereafter, H_2_O_2_ content declined to varying degrees in different treatments (Fig. 3; *p* < 0.05). Treatment with CHT, CuCl_2_ and harpin reduced H_2_O_2_ content by ~ 31%, ~ 38% and ~ 41%, respectively, in jujube fruit after storage for 7 days, compared with day 1 (Fig. 3; *p* < 0.05). However, after treatment for 14 days, H_2_O_2_ levels in postharvest jujube fruit were ordered controls > CHT > CuCl_2_ > harpin > melatonin. Application of CHT, CuCl_2_, harpin, and melatonin decreased H_2_O_2_ content in jujube fruit by ~ 24%, ~ 33%, ~ 40% and ~ 45%, respectively, after storage for 14 days, relative to controls (Fig. 3; *p* < 0.05).

### Fruit senescence, pesticide residues, and Cu content

**Figure 4:**
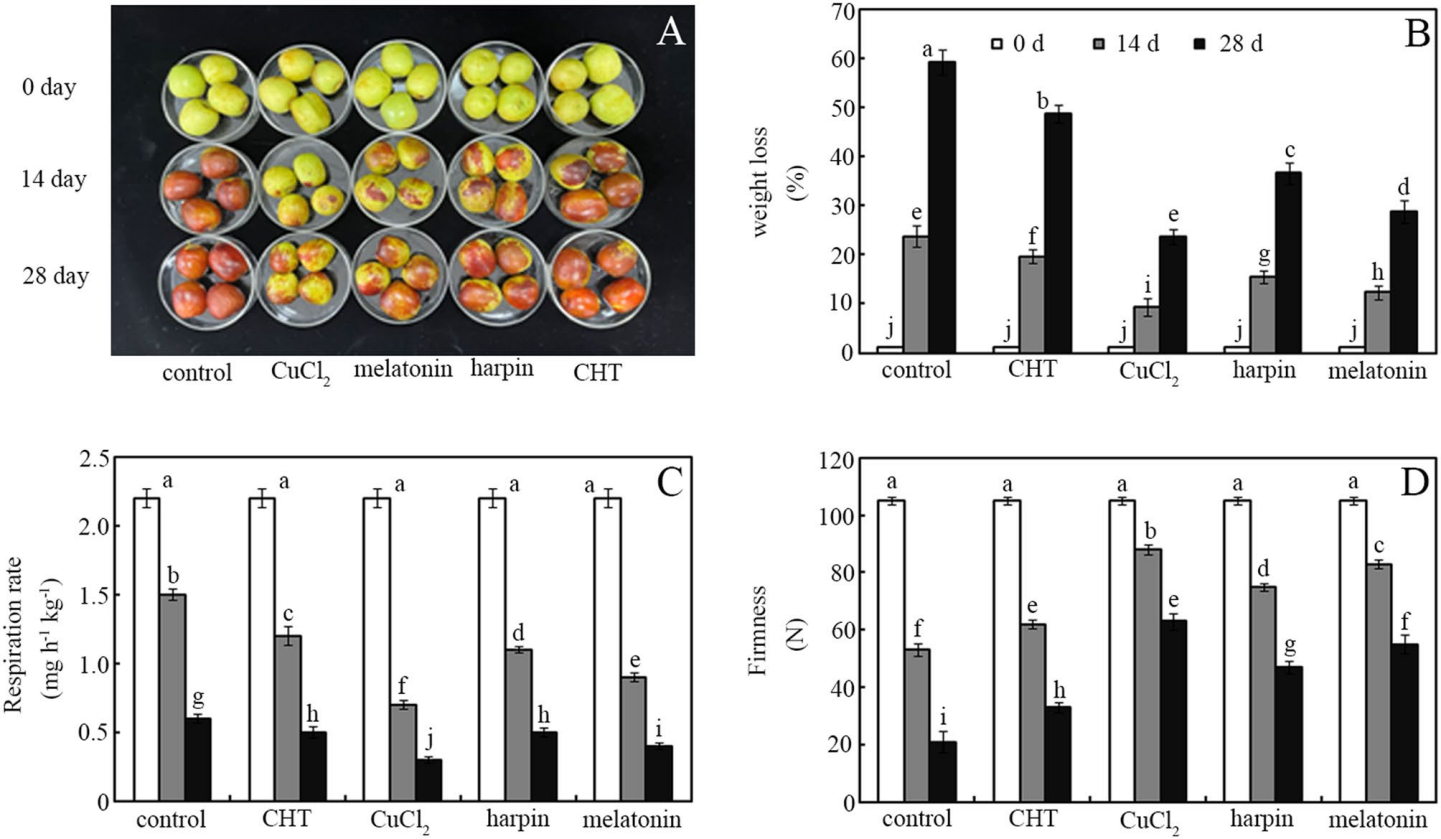
Postharvest senescence of jujube fruit. Effects of chlorothalonil, CuCl_2_, harpin and melatonin on phenotype (**A**), weight loss (**B**), respiration rate (**C**) and firmness (**D**) were monitored in jujube fruit after storage for 0, 14 and 28 days at 4 °C. Bars represent standard deviation of the mean (*n* = 3); means associated with the same letter are not significantly different (*p* < 0.05).

Compared with controls, treatment with CHT, CuCl_2_, harpin and melatonin significantly delayed jujube fruit senescence by reducing weight loss and respiration rate, and enhancing firmness during storage, to varying degrees (Fig. 4). Weight loss and respiration rate in postharvest jujube fruit were ordered controls > CHT > harpin > melatonin > CuCl_2_. Treatment with CHT, CuCl_2_, harpin, and melatonin significantly reduced weight loss in jujube fruit by ~ 18%, ~ 60%, ~ 38% and ~ 51%, respectively, after storage for 28 days, relative to controls (Fig. 4b; *p* < 0.05). Similarly, application of CHT, CuCl_2_, harpin, and melatonin decreased respiration rate in jujube fruit by ~ 20%, ~ 53%, ~ 27% and ~ 40%, respectively, after storage for 14 days, relative to controls (Fig. 4c; *p* < 0.05). However, firmness of postharvest jujube fruit was ordered CuCl_2_ > melatonin > harpin > CHT > controls. Treatment with CHT, CuCl_2_, harpin, and melatonin increased firmness of jujube fruit by ~ 57%, ~ 200%, ~ 123% and ~ 161%, respectively, after storage for 28 days, relative to controls (Fig. 4d; *p* < 0.05).

Moreover, CHT residues were reduced by ~ 38%, ~ 74%, ~ 90% and ~ 98% in jujube fruit after storage for 7, 14, 21 and 28 days, respectively, compared with day 0 (Table 2; *p* < 0.05). By contrast, Cu content was increased 256–296% in CuCl_2_-treated fruit, relative to controls (Table 2; *p* < 0.05).

**Table 2:** Pesticide residues and copper content in jujube fruit.

	Day 0	Day 7	Day 14	Day 21	Day 28
CHT	435.3 ± 24.6^a^	268.7 ± 19.4^b^	112.4 ± 8.3^c^	45.5 ± 3.2^d^	8.3 ± 0.6^e^
Cu	2.5 ± 0.2^b^	8.9 ± 0.7^a^	9.5 ± 0.4^a^	9.7 ± 0.3^a^	9.8 ± 0.5^a^

Chlorothalonil residues and copper content (mg kg^−1^ dry weight) were monitored in jujube fruit after treatment for 0 (water controls for Cu), 7, 14, 21 and 28 days at 4 °C. Means associated with the same letter are not significantly different for each line (*n* = 3; *p* < 0.05).

## Discussion

Infectious disease and senescence can severely decrease postharvest fruit nutrition quality due to antioxidant nutrient loss^26^. In the present work, the nutrition quality of postharvest jujube fruit was evaluated based on disease severity, antioxidant nutrient accumulation, and senescence level. We investigated how the four disease control agents affect postharvest quality of jujube fruit without sterilization. Compared with controls, application of CHT, CuCl_2_, harpin and melatonin significantly attenuated disease severity to varying degrees during storage (Table 1). CHT and melatonin exhibited the strongest and weakest ability, respectively, for disease resistance in plants (Table 1). This shows that organic pesticides (CHT) exhibited greater inhibitory effects on disease development in jujube fruit during storage than did inorganic (CuCl_2_) and biological (harpin) pesticides, and phytohormones (melatonin).

Reports showed that defense enzyme such as PAL and PPO, coupled with GST and GR, play different roles in regulating disease resistance^25^ and pesticide degradation in plants^13^. Interestingly, research showed that Cu^27^, harpin^25^ and melatonin^28^ can strongly stimulate PAL and PPO activities in plants, whereas CHT can profoundly enhance GST and GR activities in plants^12,13^. Thus, the effects of CHT, CuCl_2_, harpin, and melatonin on PAL, PPO, GST, and GR activities in postharvest jujube fruit were investigated (Fig. 1). Compared with controls, application of CHT, CuCl_2_, harpin and melatonin increased PAL, PPO, GST, and GR activities during storage to varying degrees (Fig. 1). However, the highest PAL and PPO activities were observed in melatonin- and harpin-treated jujube fruit during storage (Fig. 1a,b). This also suggests that CHT and CuCl_2_ inhibited disease development via a mechanism that is not closely associated with the activities of PAL and PPO, two key enzymes involved in phenolics metabolism^30^. CHT and CuCl_2_ may block disease progression by inhibiting glyceraldehyde-3-phosphate dehydrogenase^11^ or by generating acute toxic hydroxyl radicals via Fenton reaction^15^, respectively. This suggests that harpin and melatonin may enhance disease resistance by stimulating PAL and PPO activities in postharvest jujube fruit. Interestingly, the highest GST and GR activities were measured in CHT-treated fruits (Fig. 1c,d). These two enzymes play an important role in regulating xenobiotic metabolism but not disease resistance in plants^29^. This suggests that they may contribute to CHT degradation in plants. Moreover, these enzymes are closely associated with antioxidant nutrient (e.g., polyphenol and glutathione) biosynthesis and accumulation in plants^30^. Therefore, how these agents affect antioxidant accumulation in postharvest jujube fruit was subsequently investigated.

The effects of CHT, CuCl_2_, harpin, and melatonin on antioxidant (e.g., ascorbic acid, glutathione, flavonoids, and phenolics) accumulation and antioxidant capacity (evaluated by Fe^3+^ reducing power) were investigated (Fig. 2). Among these antioxidants, phenolics and flavonoids also play a key role in disease resistance^31,32^. Compared with controls, these agents drastically enhanced antioxidant accumulation in jujube fruit during storage (Fig. 2). Interestingly, the antioxidant accumulation level and total antioxidant capacity measured in postharvest jujube fruit following treatment was ordered melatonin > harpin > CuCl_2_ > CHT (Fig. 2). Melatonin exhibited the strongest stimulation of antioxidant biosynthesis in postharvest jujube fruit. This may be associated with its antioxidant properties and strong ability to activate antioxidant systems in plants^19^. Similarly, harpin and a trace amount of CuCl_2_ also induced antioxidant biosynthesis and accumulation in plants via activation of the antioxidant defense system^23,33^. Compared with controls, application of CHT only slightly enhanced antioxidant accumulation and total antioxidant capacity in postharvest jujube fruit (Fig. 2). Consistently, previous reports have shown that organically grown crops contain higher levels of antioxidant and nutrient compounds than conventionally grown crops exposed to pesticides for disease control^34,35^. Thus, the lowest total antioxidant capacity and highest disease control were monitored simultaneously in CHT-treated jujube fruit during storage (Figs. 1 and 2). This phenomenon could be partly attributed to the toxicity of CHT when binding and depleting cellular glutathione, and it can also inhibit glycolysis by binding to glyceraldehyde 3-phosphate dehydrogenase, leading to cell death^11,36,37^. However, it is also suggested that disease resistance is not closely associated with antioxidant capacity in postharvest jujube fruit. Previous reports showed that free radicals and/or reactive oxygen species (ROS) play a key role in regulating disease resistance^15,24^ and antioxidant biosynthesis in plants^38,39^. Therefore, we explored how these agents affect ROS production and accumulation in postharvest jujube fruit.

H_2_O_2_ content was determined in postharvest jujube fruit during the first 28 days (Fig. 3). The results showed that CHT, CuCl_2_ and harpin rapidly induced H_2_O_2_ accumulation in jujube fruit after treatment for 1 day (Fig. 3). Moreover, the results show that CHT, CuCl_2_ and harpin play a role, as oxidant-like inducers^32^, in stimulating antioxidant biosynthesis and accumulation via ROS production in jujube fruit during storage (Fig. 3). However, more evidence is required to determine the detailed mechanism. By contrast, melatonin inhibited H_2_O_2_ overproduction, and no peak was observed during the whole storage stage (Fig. 3). However, the highest antioxidant accumulation was measured in melatonin-treated jujube fruit (Fig. 2). This indicates that melatonin, which acts as a phytohormone, can efficiently regulate antioxidant biosynthesis and accumulation via a different mechanism^19–21^.

Antioxidants (e.g., ascorbic acid and glutathione) also play a key role in delaying fruit and vegetable senescence during storage^39^. Therefore, we next investigated how these agents affect postharvest jujube fruit senescence (Fig. 4). Compared with controls, treatment with these agents delayed jujube fruit senescence (evaluated by weight loss, respiration rate and firmness) to varying degrees (Fig. 3). The senescence-delaying effects of these agents were ordered CuCl_2_ > melatonin > harpin > CHT (Fig. 4). This order is similar to that of antioxidant accumulation and antioxidant capacity: melatonin > harpin > CuCl_2_ > CHT (Fig. 2). This shows that the senescence-delaying effects are closely associated with antioxidant accumulation in postharvest jujube fruit (Figs. 2 and 4). This is consistent with published data^6,7,9,23^, which showed that antioxidant capacity made a key contribution to delaying senescence of postharvest fruit and vegetables. However, CuCl_2_ rather than melatonin exhibited the greatest senescence-delaying effects (Figs. 2 and 4; *p* < 0.05). This phenomenon could be partly attributed to the inhibitory effects of copper-induced oxidative stress on aquaporin, a key channel for water permeability in plants^40^. Consistently, the lowest decline in weight loss was observed in CuCl_2_-treated jujube fruit during storage (Fig. 4b). Compared with CHT, melatonin and harpin drastically delayed postharvest fruit senescence to varying degrees (Fig. 4). One plausible explanation is their ability to induce antioxidant accumulation in plants^19,23^. However, CHT exhibited the lowest ability to delay postharvest fruit senescence among the four agents (Fig. 4). This also showed that antioxidant capacity but not disease control ability makes a greater contribution to delaying postharvest senescence in jujube fruit.

Among the four disease control agents, the pesticide CHT exhibited the strongest inhibitory effects on disease development, but this was coupled with the lowest antioxidant content and ability to delay senescence in postharvest jujube fruit. Moreover, Table 2 shows that pesticide (CHT) residues were detected in jujube fruit even after storage for 4 weeks^14^. This poses a great threat to human health and the environment. By contrast, melatonin and harpin achieved higher antioxidant levels but exerted lower inhibitory effects on disease development. Interestingly, CuCl_2_ application not only drastically reduced disease severity but also induced antioxidant biosynthesis and accumulation^41^. In addition, CuCl_2_ treatment enhanced copper accumulation (~ threefold) in jujube fruit, relative to controls (Table 2). It is known that trace amounts of copper are required for human health^42^. Thus, application of CuCl_2_ may perform better than applying other agents such as CHT, harpin, and melatonin in terms of simultaneously controlling disease, delaying senescence, and increasing antioxidant accumulation in postharvest jujube fruit without sterilization.

In conclusion, the tested agents enhanced disease control and bioactive compound accumulation, and delayed senescence in postharvest jujube fruit to varying degrees. Among the disease control agents tested, CHT performed best for disease control, but worst for delaying senescence and promoting antioxidant accumulation. By contrast, melatonin performed best for enhancing antioxidant capacity, but worst for disease control. However, delaying jujube fruit senescence and inhibition of disease development could be efficiently achieved by CuCl_2_ treatment. This showed that antioxidant capacity was closely associated with postharvest senescence, but not closely associated with disease control ability. Copper, the key component of the traditional pesticide Bordeaux mixture^15^, was tested for its ability to efficiently control disease development, increase antioxidant accumulation, and delay senescence in postharvest jujube fruit. The results provide a simple method for improving postharvest jujube fruit quality under low temperature (4 °C) without sterilization. This phenomenon could be partly attributed to the different roles of these agents in regulating the content of H_2_O_2_, which is closely associated with antioxidant content, postharvest senescence, and disease development in plants. Future studies should focus on the molecular mechanism underpinning this phenomenon.

## Materials and methods

All local, national or international guidelines and legislation were followed during the course of this study.

### Reagent preparation

Harpin protein and chlorothalonil (Daconil-2787) were obtained from Haibos Biotech Company (Chengdu, China) and Macklin Biochemistry & Technique Company (Shanghai, China), respectively. Harpin (30 mg L^−1^) and CHT (10 mM) were used at concentrations recommended in the respective manuals.

### Experiment design

Jujube (*Ziziphus jujuba* Mill. cv. Dongzao) fruits were harvested from a commercial orchard in Luoyang, Henan province, China, at the commercial maturity stage (80 days old with light green peel after full bloom)^43^. Fruits without visible defects were chosen based on uniform shape and appearance. Postharvest jujube fruits without sterilization were divided into five groups (water control, CHT, harpin, CuCl_2_, and melatonin) and used in subsequent experiments. Treatments were performed under low temperature conditions (4 °C, 70% humidity) as follows:

*Step 1*: fruit were immersed in distilled water (controls), chlorothalonil (10 mM), harpin (30 mg L^−1^), CuCl_2_ (0.5 g L^−1^) or melatonin (0.1 mM) for 2 h;

*Step 2*: treated fruit were washed with distilled water and dried in air at 25 °C for 2 h;

*Step 3*: treated fruit were stored at 4 °C and 70% relative humidity for up to 28 days.

One hundred samples were used each week to evaluate weight loss, firmness, respiration rate and disease index. Additionally, other samples of jujube fruits were stored for 7, 14, 21 and 28 days at 4 °C prior to analysis of CHT and copper accumulation, ascorbic acid content, glutathione level, total phenolics and flavonoids content, total antioxidant capacity, and measuring enzyme activities for PAL, PPO, GR, and GST activities.

Herein, only edible fruit tissues (including flesh and peel) were collected for parameter analysis. For each assay, three replicates were performed for each treatment, each including 20–30 fruits.

### Copper assay

Jujube fruits were collected, washed, air-dried, and ground into powder with a mortar and pestle. For heavy metal extraction, digestion tubes were thoroughly acid washed and dried^8^. Dry powdered sample (1 g) was placed in a digestion tube and 10 mL of HNO_3_, HClO_4_ and H_2_SO_4_ (5:1:1) were added and incubated for 12 h. The tubes were then placed in a digestion block at 80 °C for 1 h, and the temperature was slowly raised to 120–130 °C. When digestion was completed, the solutions were cooled, filtered, and diluted to 100 mL with double-deionized water. Cu in filtrates was assayed using an Analyst 700 atomic absorption spectrometer (Perkin Elmer, USA).

### Pesticide residue assay

Pure chlorothalonil was obtained from Macklin Biochemistry & Technique Company (Shanghai, China). Pesticide residues were extracted and determined in 5 g of chopped fruit tissues^7^. Jujube fruits combined with petroleum ether (PE) and anhydrous sodium sulphate (ASS) were homogenized in a high-speed disperser (12,000 × *g* for 5 min). A Büchner funnel (7 cm) containing 10 g of ASS was used for filtration of the fruit mixture, and 50 mL of redistilled PE was used to wash the filter cake three times. The filtrates were mixed in a flat-bottomed flask (0.5 L) and dried with an N_2_ stream. Chlorothalonil was dissolved in redistilled PE, and 5 mL of solution was subjected to quantitative analysis by a gas chromatography instrument (GC-14C, Shimadzu, Japan) equipped with a phosphorus filter and a flame photometric detector.

### Disease index assay

The disease index was used to evaluate the development of disease resulting from natural infection. It was assessed by monitoring the growth of visible pathogen lesions on the jujube fruit surface as described previously^44^. Disease index is divided into five levels: grade 0, no lesions; grade 1, some lesions; grade 2, lesion area < 25%; grade 3, lesion area 25–50%; grade 4, lesion area > 50%. Disease index was assessed by measuring the lesion area on each fruit pericarp and calculated using the equation:

$${\text{Disease}}\;{\text{index}} = \left( {{\text{disease}}\;{\text{scale}} \times {\text{number}}\;{\text{of}}\;{\text{fruits}}\;{\text{within}}\;{\text{the}}\;{\text{corresponding}}\;{\text{scale}}} \right)/\left( {{\text{total}}\;{\text{number}}\;{\text{of}}\;{\text{fruits}} \times {\text{maximum}}\;{\text{severity}}\;{\text{scale}}} \right) \times {\text{1}}00\% .$$

### Weight loss, firmness, and respiration rate analyses

Weight loss was evaluated by weighing each jujube fruit before and after the storage period, and presented as the percentage weight loss compared to initial weight. Firmness was measured using a GY-3 pressure tester (Aidebao Instrument Co. Ltd, Leqing, China) equipped with an 8 mm diameter probe. Decay incidence is the number of fruits showing decay symptoms relative to the total number of fruits in each treatment (expressed in %). Respiration rate was estimated using a previously described method^45^ with some modifications. In each treatment, 10 jujube fruits (water, chlorothalonil, harpin, CuCl_2_ or melatonin) were randomly sampled and sealed in a glass container with 0.02 L 0.4 M NaOH at room temperature for 1 h. Next, 0.01 L saturated BaCl_2_ and three drops of phenolphthalein were added, and the solution was titrated with 0.1 M oxalic acid until the red color disappeared. The respiration rate of samples is expressed as mg kg^−1^ h^−1^ CO_2_.

### Antioxidant measurement

The titrimetric method with 2,6-dichloro-phenol-indophenol (2,6-DPI) was used to assess the ascorbic acid content^7^. Briefly, 1 g of homogenized fresh jujube fruit was mixed with 20 mL of 2% oxalic acid. The mixture was homogenized, diluted to 0.1 L with 2% oxalic acid, and filtered. Next, 10 mL of filtered solution was titrated with 0.01% 2,6-DPI solution. The endpoint was considered reached when the solution had been pink in color for 15 s. Calibration of the 2,6-DPI solution was performed using a 0.05% ascorbic acid solution. Results are expressed as mg ascorbic acid equivalents per g of fresh weight (mg g^−1^).

Glutathione was determined by an enzymatic cycling assay described previously^46^. Oxidized glutathione (GSSG) was measured after removal reduced glutathione (GSH) by 2-vinylpyridine derivatization. GSH was determined by subtraction of GSSG from total glutathione (GSH + GSSG).

Total phenolics were measured using the Folin-Ciocalteu reagent method^47^; the absorbance was recorded at 760 nm using a UV–Vis spectrophotometer (Shimadzu, Kyoto, Japan) and results expressed as gallic acid equivalents (mg g^−1^ of dry weight).

The total flavonoid content was determined by the aluminum chloride colorimetric method^48^ using catechin as a standard, and expressed as mg of catechin equivalent (CE) per kg of dry weight.

Total antioxidant capacity assays were performed described previously^49^. Fruits were ground to powder in liquid N_2_ using a mortar and pestle, 5 g of fruit tissue powder was transferred to 1 L of 80% (w/v) methanol–water solution, and incubated at room temperature for 2 h in the dark. Extracts were filtered, filtrates from each replicate were pooled, and solvent was removed under vacuum at 45 °C using a rotary evaporator. Crude extracts were then stored in a desiccator at 4 °C for subsequent total antioxidant capacity analysis using the ferric reducing ability of plasma (FRAP) assay. FRAP reagent comprised 10:1:1 (v/v) 100 mM acetate buffer (pH 3.6), 20 mM FeCl_3_ solution and 10 mM 2,4,6-tripyridyl triazine solution in 40 mM HCl. FRAP reagent was prepared and warmed to 37 °C in a water bath just before use. Samples (50 µL) were added to 1.5 mL of FRAP reagent and the absorbance of the reaction mixture was recorded at 593 nm after 5 min using a UV–Vis spectroscopy instrument (Shimadzu). A standard curve was constructed using FeSO_4_ solution and results expressed as mM Fe(II) g^−1^ dry weight of jujube fruit.

### Hydrogen peroxide assay

Hydrogen peroxide (H_2_O_2_) accumulation in jujube fruits was measured using the oxidation Xylenol Orange assay^50^, which is based on the oxidation of Fe(II) by peroxide, followed by colorimetric detection of the reaction of Fe(III) with the sodium salt of Xylenol Orange. A 1 mL sample of assay reagent (25 mM FeSO_4_ and 25 mM (NH_4_)_2_SO_4_, dissolved in 2.5 M H_2_SO_4_) was added to 100 mL of 125 μM Xylenol Orange and 100 mM sorbitol. Fruit samples were ground and centrifuged at 6,000 g for 15 min. The supernatant (100 μL) was added to 1 mL of Xylenol Orange reagent. After 30 min of incubation, absorbance by the Fe(III)–Xylenol Orange complex was recorded at 560 nm.

### Defense enzyme activity assays

Fruit tissue was extracted using potassium phosphate buffer (50 mM, pH 7.5). Homogenates were centrifuged at 12,000 g for 30 min and the supernatant was used for enzyme activity assays. PAL (EC 4.3.1.5) activity was determined as described previously^51^. Samples containing 0.1 mL of enzyme extract were incubated with 1.2 mL of 0.1 M borate buffer (pH 8.8) and 1.5 mL of 12 M L-phenyl alanine in the same buffer for 30 min at 30 °C. The reaction was stopped by addition of 1 M trichloroacetic acid, and after incubation for 5 min at 37 °C, the absorbance was recorded at 290 nm. Enzyme activity was expressed as mmol trans-cinnamic acid min^−1^ mg^−1^ protein.

PPO (EC 1.14.18.1) activity was measured s described previously^52^. The reaction mixture consisted of 1.5 mL of 0.1 M sodium phosphate buffer (pH 6.5) and 100 μL of enzyme extract. To start the reaction, 0.2 mL of 0.01 M catechol was added and activity was measured as the change in absorbance at 495 nm min^−1^ mg^−1^ protein.

GST (EC 2.5.1.18) activity was determined using a GST Colorimetric Activity Assay Kit (Jiancheng BioCo., Nanjing, China). Reactions contained 50 mM potassium phosphate (pH 6.5) at 25 °C, aliquots of enzyme extract, 5 mM GSH, 0.4 mM 1-chloro-2,4-dinitrobenzene (CDNB), and 1% (v/v) ethanol in a final volume of 1 mL. Reactions were initiated by addition of CDNB substrate in ethanol. Enzymatic formation of 2,4-dinitrophenyl-S-glutathione at 340 nm (E = 9.6 mM^−1^ cm^−1^) was monitored for 5 min and corrected using non-enzymatic controls^7^.

GR (EC 1.6.4.2) activity was measured based on the rate of decrease in the absorbance of NADPH at 340 nm^53^.

Soluble protein was determined using the Bradford method^54^ using bovine serum albumin as standard. All spectrophotometric analyses were conducted on a Shimadzu UV2410 PC spectrophotometer (Shimadzu).

### Data analysis

All experiments were conducted in a completely randomized design, with three replicates per treatment. All data were analyzed using Duncan’s multiple range test with SPSS 13.0 software (IBM Corp., Armonk, NY, USA) and *p* ˂0.05 was considered statistically significant.

## Data Availability

All data analyzed during this study are included in the published article. Raw data are available on reasonable request from the corresponding author (yueyueli2020@163.com).
